# Supplementary material for: Effects of Short-Term Nitrogen Addition on Soil Fungal Community Increase with Nitrogen Addition Rate in an Alpine Steppe at the Source of Brahmaputra
Source: Microorganisms. 2023 Jul 25;11(8):1880. doi: 10.3390/microorganisms11081880 (PMC10458649; doi:10.3390/microorganisms11081880)
Supplement: Supplementary file 1 [file microorganisms-11-01880-s001.zip › microorganisms-2473774-supplementary.pdf]

microorganisms

MDPI

Article

Effects of short-term nitrogen addition on soil fungal community increase with nitrogen addition rate in an alpine steppe at the source of Brahmaputra

Shaolin Huang [1,2], Chengqun Yu [1,*], Gang Fu [1], Wei Sun [1], Shaowei Li [1], Fusong Han [3] and Jianyu Xiao [1,2]

[1] Lhasa Plateau Ecosystem Research Station, Key Laboratory of Ecosystem Network Observation and Modeling, Institute of Geographic Sciences and Natural Resources Research, Chinese Academy of Sciences, Beijing 100101, China
[2] University of Chinese Academy of Sciences, Beijing 100049, China
[3] College of Urban and Environment Sciences, Hunan University of Technology, Hunan 412007, China
* Correspondence: yucq@igsnrr.ac.cn

Measuring methods of soil physicochemical property

Soil organic carbon was determined by potassium dichromate volumetric method ($K_2Cr_2O_7$-H_2SO_4). Total nitrogen was determined by Kjeldahl nitrogen determination method. Soil ammonium nitrogen and nitrate nitrogen were determined by indophenol blue colorimetric method and hydrazyl sulfate method respectively. Soil total phosphorus was determined by molybdenum-antimony resistance colorimetric method, and available phosphorus was determined by ammonium bicarbonate leaching - molybdenum-antimony resistance colorimetric method. And pH value was determined by pH meter method. The moisture content was determined by drying. Soil urease was determined by indophenol blue colorimetry. Soil alkaline phosphatase was determined by colorimetry. Soil catalase was determined by potassium permanganate titration. Soil saccharase and cellulase were determined by 3, 5-dinitrosalicylic acid colorimetry.

High-Throughput Sequencing

We used 0.50 g soil to extract DNA. The concentration and purity were measured using the NanoDrop One (Thermo Fisher Scientific, MA, USA). The barcoded primers ITS1F (5′-CTTGGTCATTTAGAGGAAGTAA-3′) and ITS2R (5′-GCTGCGTTCTTCATCGATGC-3′) were used to amplify the ITS1 and ITS2 regions of the soil fungal community. PCR reactions, containing 25 μL 2x Premix Taq (Takara Biotechnology, Dalian Co. Ltd., China), 1 μL each primer (10 μM), and 3 μL DNA (20 ng/μL) template in a volume of 50 μL, were amplified via thermocycling: 5 min at 94°C for initialization; 30 cycles of 30 s denaturation at 94°C, 30 s annealing at 52°C, and 30 s extension at 72°C; followed by 10 min final elongation at 72°C. The length and concentration of the PCR product were detected using 1% agarose gel electrophoresis. Samples with a bright main strip between could be uesd for further experiments. PCR products were mixed in equidensity ratios according to the GeneTools Analysis Software (Version4.03.05.0, SynGene). Then, the mixture of PCR products was purified using the E.Z.N.A. Gel Extraction Kit (Omega, USA). Sequencing libraries were generated using the NEBNext® Ultra™ II DNA Library Prep Kit for Illumina® (New England Biolabs, MA, USA). At last, the library was sequenced on an Illumina Nova6000 platform and 250 bp paired-end reads were generated.

Supplementary Figures

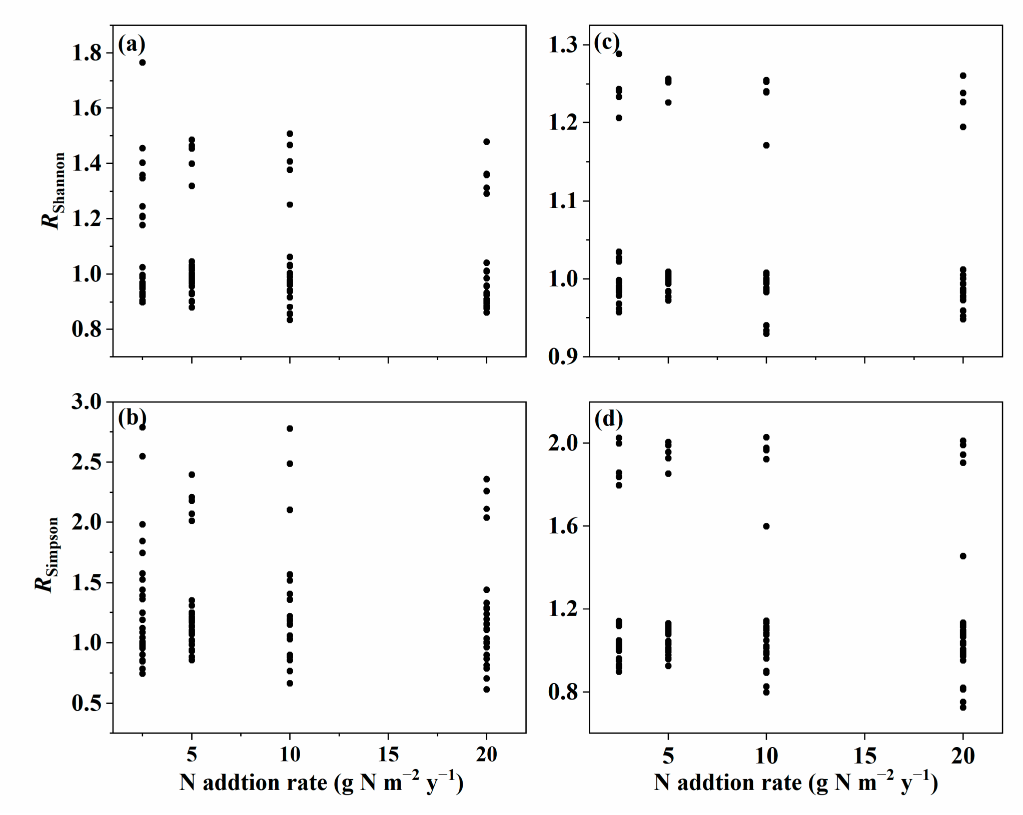

Figure S1. Relationships between nitrogen addition rate and the effect size of nitrogen addition on species α-diversity of soil fungi in an alpine steppe. Surface soil: (a) Shannon; (b) Simpson. Subsurface soil: (c) Shannon; (d) Simpson.

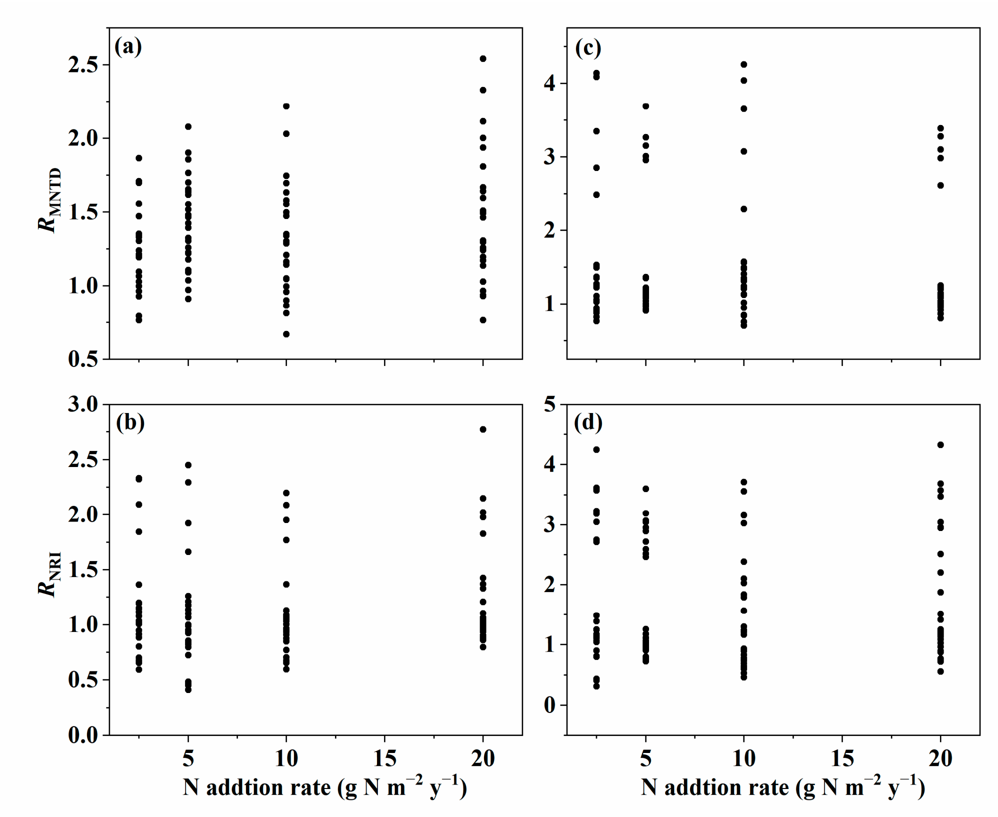

Figure S2. Relationships between nitrogen addition rate and the effect size of nitrogen addition on phylogenetic α-diversity of soil fungi in an alpine steppe. Surface soil: (a) MNTD; (b) NRI. Subsurface soil: (c) MNTD; (d) NRI.

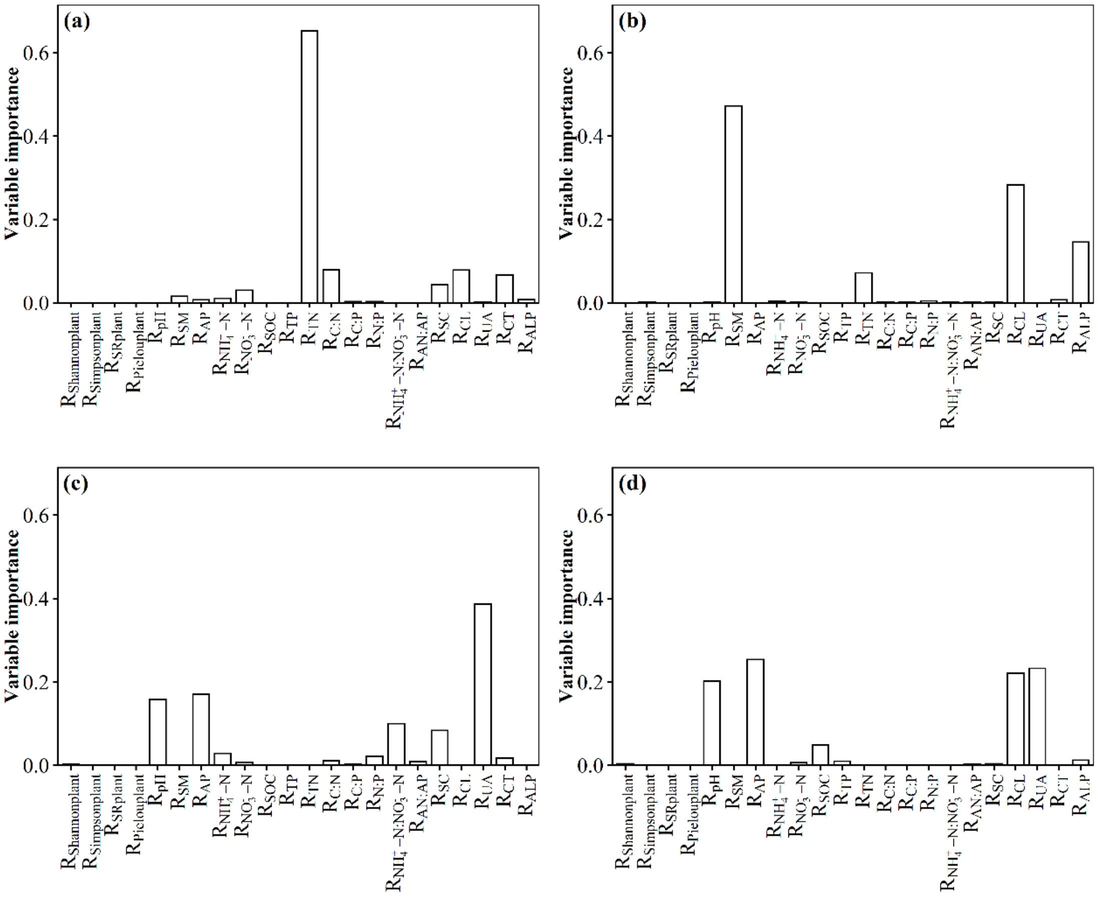

Figure S3. Relative contribution of observed plant and soil characteristics to effect size of nitrogen addition on species α-diversity of soil fungi in an alpine steppe. Surface soil: (a) Shannon; (b) Simpson. Subsurface soil: (c) Shannon; (d) Simpson.

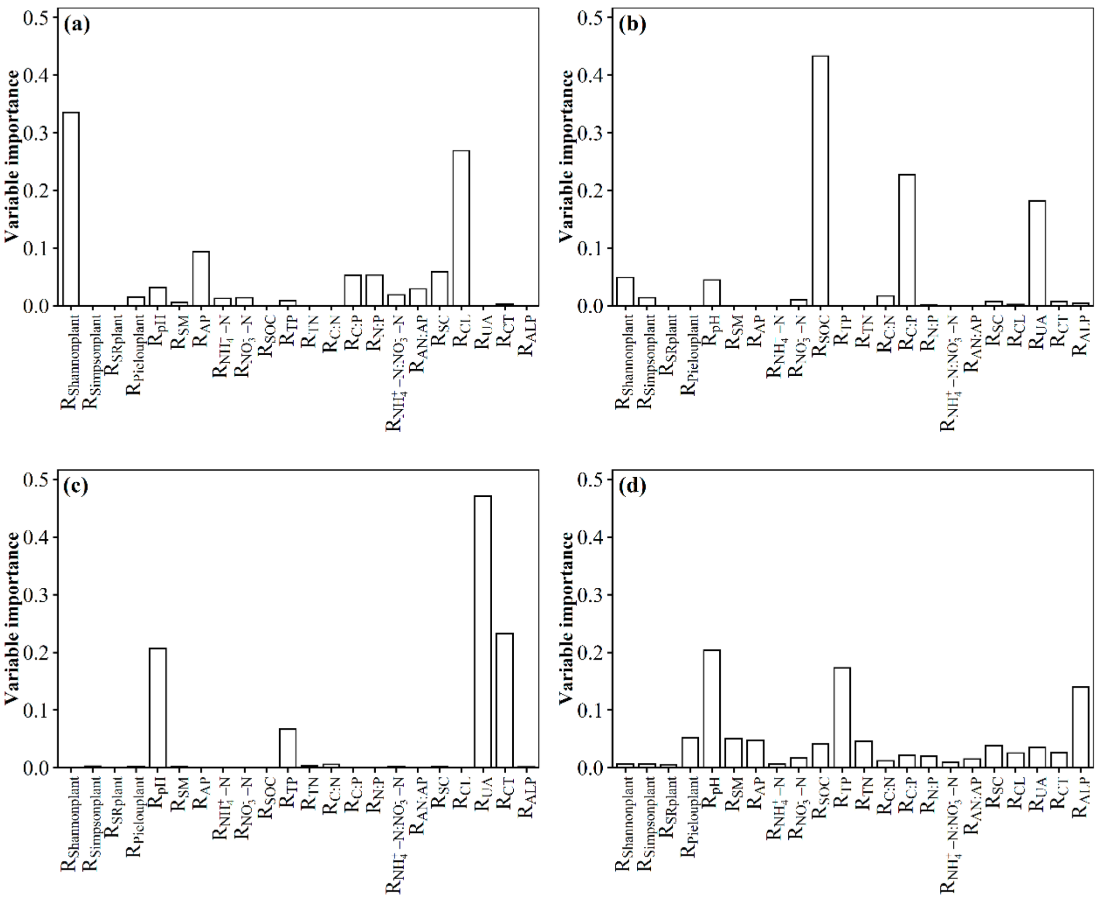

Figure S4. Relative contribution of observed plant and soil characteristics to effect size of nitrogen addition on phylogenetic α-diversity of soil fungi in an alpine steppe. Surface soil: (a) MNTD; (b) NRI. Subsurface soil: (c) MNTD; (d) NRI.

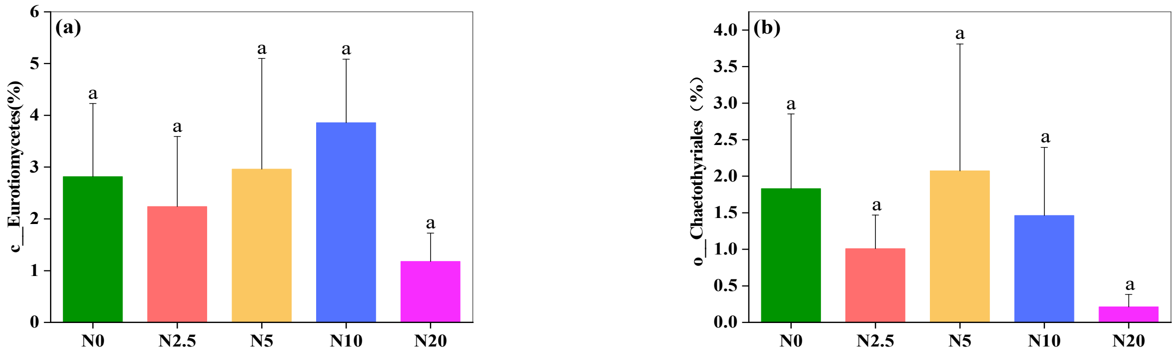

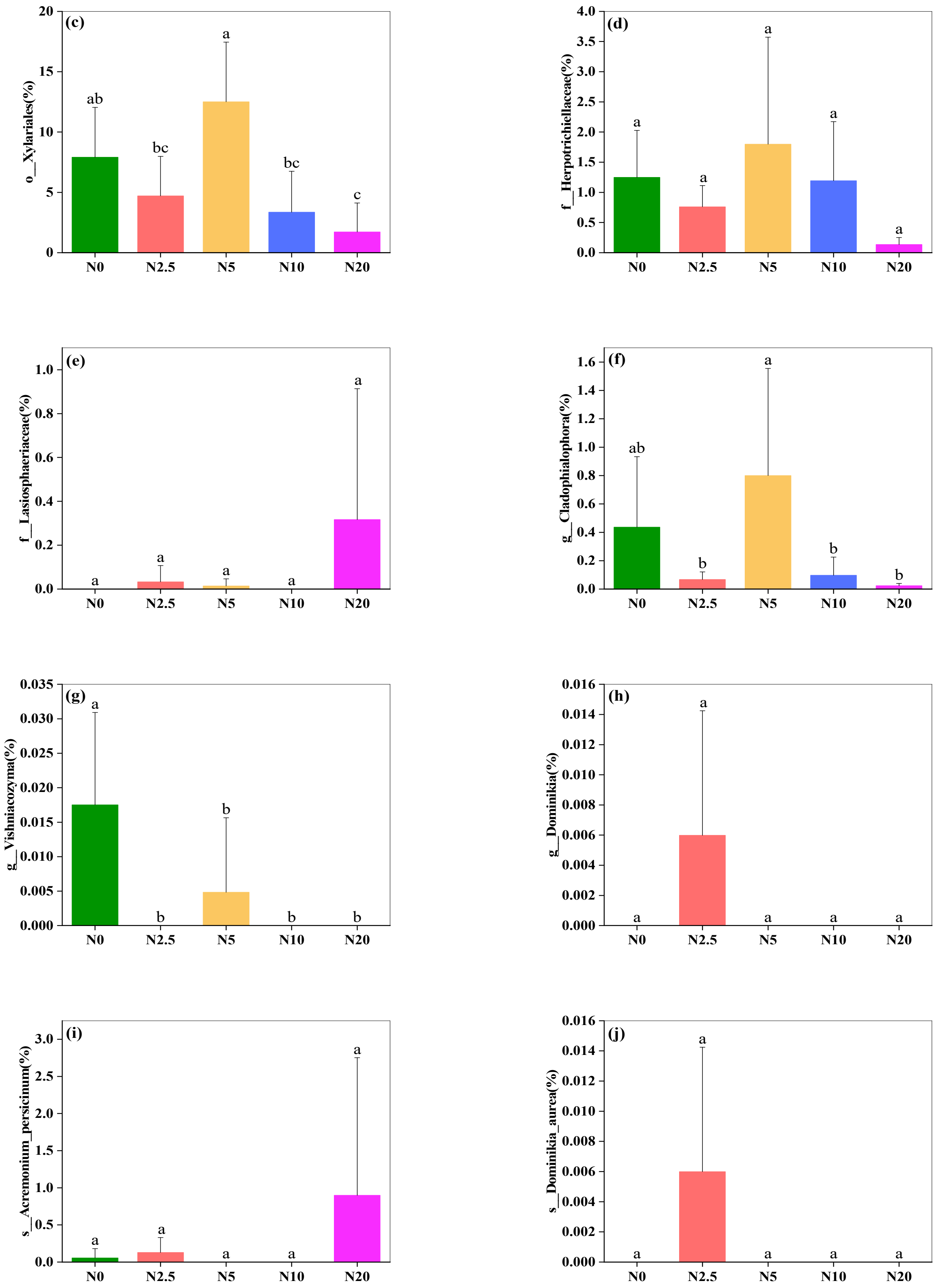

Figure S5. Duncan multi-comparison of soil fungal community in surface soil at the class, order, family, genus, and species level along the N addition gradient based on LEFSe analysis. (Different letters indicate significant differences between nitrogen addition treatments.)

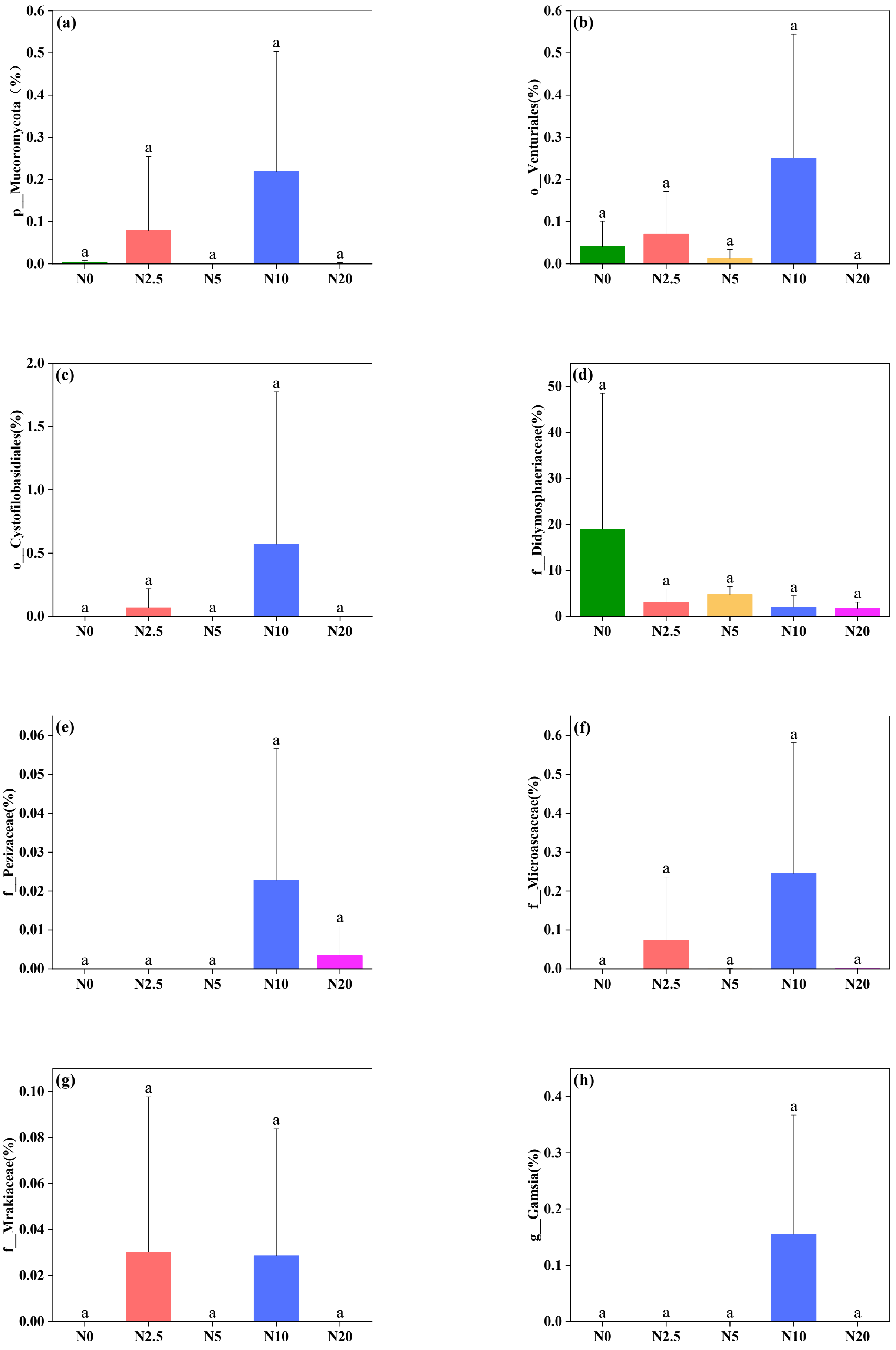

(a)
p__Mucoromycota（%）
0.0
0.1
0.2
0.3
0.4
0.5
0.6
a
N0
N2.5
N5
N10
N20
(b)
o__Venturiales(%)
(c)
o__Cystofilobasidiales(%)
0.5
1.0
1.5
2.0
(d)
f__Didymosphaeriaceae(%)
0
10
20
30
40
50
(e)
f__Pezizaceae(%)
0.00
0.01
0.02
0.03
0.04
0.05
0.06
(f)
f__Microascaceae(%)
(g)
f__Mrakiaceae(%)
0.02
0.04
0.06
0.08
0.10
(h)
g__Gamsia(%)

Figure S6. Duncan multi-comparison of soil fungal community in subsurface soil at the phylum, order, family, and genus level along the N addition gradient based on LEFSe analysis. (Different letters indicate significant differences between nitrogen addition treatments.)

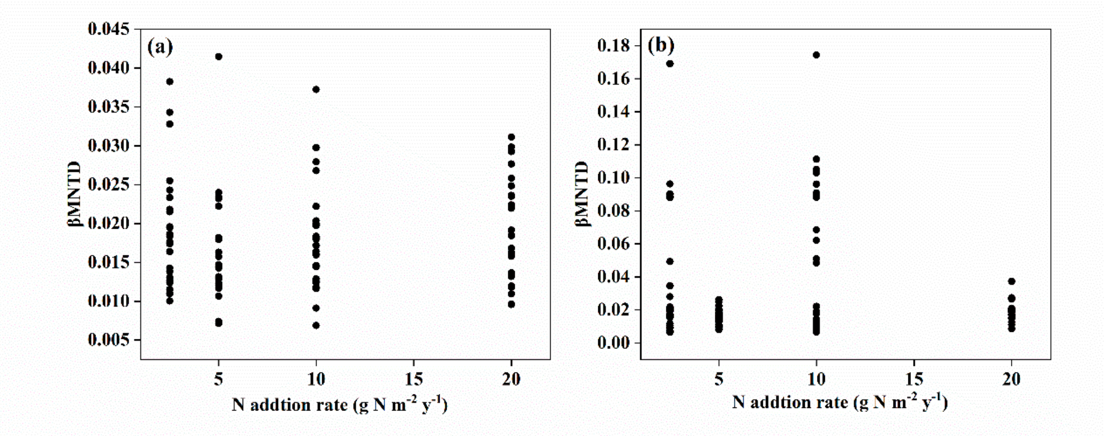

Figure S7. Change in effect size of nitrogen addition on soil fungal phylogenetic β-MNTD in surface soil (a) and subsurface soil (b) along the N addition gradient in an alpine steppe.

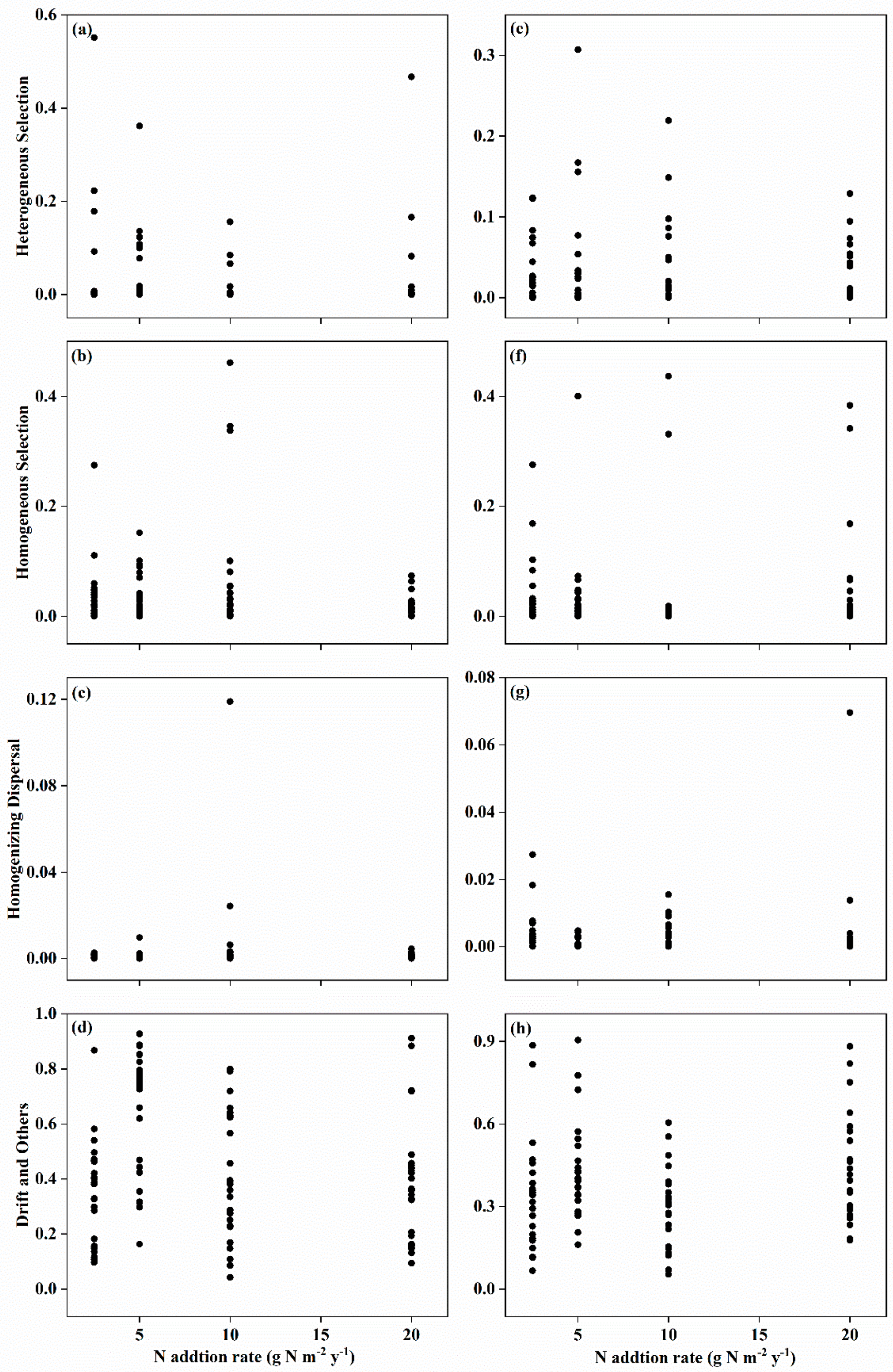

Figure S8. Change in effect size of nitrogen addition in an alpine steppe. Surface soil: (a) Heterogeneous Selection; (b) Homogeneous Selection; (c) Homogenizing Dispersal; (d) Drift and Others. Subsurface soil: (e) Heterogeneous Selection; (f) Homogeneous Selection; (g) Homogenizing Dispersal; (h) Drift and Others.

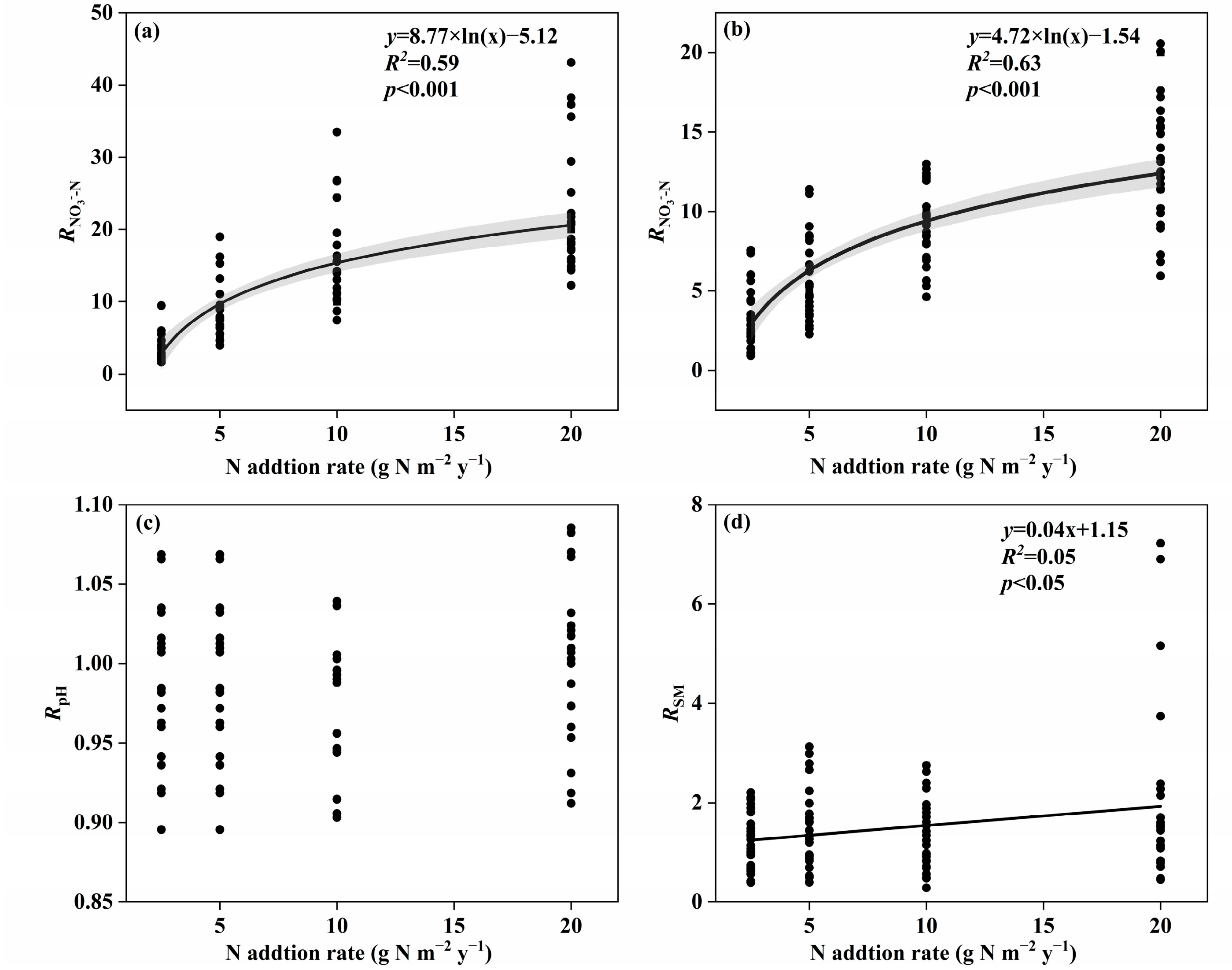

Figure S9. Change in effect size of nitrogen addition in an alpine steppe: (a) soil NO_3^--N in surface soil, (b) soil NO_3^--N in subsurface soil, (c) pH in subsurface soil, and (d) SM in surface soil. NO_3^--N: nitrate nitrogen; SM: soil moisture.

Supplementary Tables

Table S1. Pearson correlation analyses between species α-diversity and soil and plant variables.

Variables	Surface					Subsurface				
	R_{OTUs}	R_{ACE}	R_{Chao1}	$R_{Shannon}$	$R_{Simpson}$	R_{OTUs}	R_{ACE}	R_{Chao1}	$R_{Shannon}$	$R_{Simpson}$
$R_{Shannonplant}$	0.28**	0.38***	0.39***	0.04	0.07	0.08	0.23*	0.13	-0.06	-0.07
$R_{Simpsonplant}$	0.31**	0.40***	0.42***	0.04	0.07	0.1	0.23*	0.14	-0.1	-0.11
$R_{SRplant}$	0.12	0.22*	0.21*	0.06	0.06	-0.05	0.13	-0.01	0.05	0.04
$R_{Pielouplant}$	0.32**	0.38***	0.42***	0.05	0.07	0.07	0.1	0.06	-0.18+	-0.16
R_{pH}	0.10	-0.06	0.01	0.24*	0.32	0.52***	0.31**	0.32**	0.45***	0.43***
R_{SM}	-0.50***	-0.24*	-0.28**	-0.28**	-0.34	-0.47***	-0.13	-0.24*	0.05	0.06
R_{AP}	0.05	0.06	0.1	-0.09	-0.04	0	0.31**	0.29**	-0.25*	-0.31**
$R_{NH_4^+\text{-}N}$	0.04	0.09	0.09	-0.15	-0.1	0.07	0.17+	0.2*	-0.08	-0.07
$R_{NO_3^-\text{-}N}$	-0.44***	-0.36***	-0.31**	-0.32**	-0.26	-0.06	-0.07	0.03	0	0.1
R_{SOC}	-0.17	-0.01	-0.08	-0.44***	-0.51	-0.22*	0.17+	0.08	-0.12	-0.09
R_{TP}	-0.46***	-0.11	-0.23*	-0.3**	-0.39	-0.35***	0.07	-0.07	-0.05	0
R_{TN}	-0.22*	0.01	-0.08	-0.49***	-0.56	-0.24*	0.18+	0.07	-0.05	0
$R_{C:N}$	-0.02	-0.18+	-0.12	-0.03	-0.07	0.08	-0.18+	-0.07	-0.02	-0.05
$R_{C:P}$	0.11	0.03	0.05	-0.34***	-0.36	-0.08	0.15	0.15	-0.13	-0.11
$R_{N:P}$	0.10	0.06	0.06	-0.38***	-0.39	-0.13	0.23*	0.18+	-0.13	-0.1
$R_{NH_4^+\text{-}N:NO_3^-\text{-}N}$	0.20*	0.24*	0.23*	-0.09	-0.04	0.22*	0.39***	0.37***	-0.08	-0.15
$R_{AN:AP}$	-0.05	0	0.03	-0.1	-0.05	0.06	0.03	0.08	-0.02	0.01

R_{SC}	-0.15	0.11	0.03	-0.27**	-0.41	-0.07	0.31**	0.21*	-0.03	-0.02
R_{CL}	-0.19+	-0.37***	-0.36***	-0.27**	-0.22	-0.36***	-0.62***	-0.53***	-0.28**	-0.27**
R_{UA}	-0.26**	-0.18+	-0.18+	-0.35***	-0.37	-0.21*	0.16	0.05	0.18+	0.21*
R_{CT}	0.29**	0.45***	0.42***	-0.21*	-0.27	0.52***	0.76***	0.71***	-0.08	-0.12
R_{ALP}	0.24*	0.28**	0.25*	-0.3**	-0.33	0.03	0.3**	0.27**	-0.16	-0.08

Note: "*", "**", and "***" means $p < 0.05$, $p < 0.01$, and $p < 0.001$, respectively.

Table S2. Pearson correlation analyses between phylogenetic α-diversity and soil and plant variables.

Variables	Surface					Subsurface				
	R_{PD}	R_{MPD}	R_{MNTD}	R_{NRI}	R_{NTI}	R_{PD}	R_{MPD}	R_{MNTD}	R_{NRI}	R_{NTI}
$R_{Shannon_{plant}}$	0.28**	0.01	0.09	0.09	-0.05	0.11	-0.08	-0.13	-0.01	0.00
$R_{Simpson_{plant}}$	0.31**	0.01	0.07	0.10	-0.04	0.12	-0.11	-0.16	-0.02	-0.03
$R_{SR_{plant}}$	0.12	0.03	0.15	0.08	-0.06	-0.01	0.03	-0.02	0.06	0.08
$R_{Pielou_{plant}}$	0.31**	0.01	0.01	0.09	0.02	0.07	-0.19	-0.17	-0.04	-0.12
R_{pH}	0.10	0.31**	0.02	0.20*	0.25*	0.48**	0.38**	0.35**	0.59**	0.52**
R_{SM}	-0.50**	-0.23*	0.08	-0.34**	-0.31**	-0.44**	0.12	0.16	-0.43**	-0.22*
R_{AP}	0.05	-0.15	-0.36**	0.10	0.14	0.00	-0.22*	-0.29**	-0.37**	-0.21*
$R_{NH_4^+\text{-N}}$	0.06	-0.18	-0.06	0.00	-0.13	0.06	-0.09	-0.13	0.12	0.09
$R_{NO_3^-\text{-N}}$	-0.43**	-0.31**	-0.06	-0.17	-0.29**	-0.07	0.07	0.09	0.06	0.10
R_{SOC}	-0.18	-0.51**	-0.34**	-0.36**	-0.31**	-0.16	-0.02	0.05	-0.49**	-0.44**
R_{TP}	-0.48**	-0.22*	0.03	-0.44**	-0.33**	-0.36**	0.07	0.08	-0.52**	-0.31**
R_{TN}	-0.23*	-0.52**	-0.30**	-0.45**	-0.39**	-0.20	0.06	0.10	-0.48**	-0.39**
$R_{C:N}$	-0.01	-0.23*	-0.21*	0.17	0.09	0.14	-0.05	0.05	0.05	-0.09
$R_{C:P}$	0.09	-0.47**	-0.41**	-0.16	-0.16	0.02	-0.07	0.02	-0.36**	-0.40**
$R_{N:P}$	0.09	-0.48**	-0.38**	-0.22*	-0.22*	-0.04	-0.06	0.00	-0.41**	-0.41**
$R_{NH_4^+\text{-N}:NO_3^-\text{-N}}$	0.22*	-0.12	-0.03	0.04	-0.11	0.24*	-0.15	-0.20*	0.17	0.06
$R_{AN:AP}$	-0.03	-0.11	0.05	0.01	-0.15	0.07	-0.04	-0.02	0.22*	0.12
R_{SC}	-0.18	-0.31**	-0.08	-0.38**	-0.30**	-0.01	0.03	0.07	-0.34**	-0.33**
R_{CL}	-0.15	-0.24*	-0.05	-0.16	-0.19	-0.37**	-0.29**	-0.25*	-0.07	-0.11
R_{UA}	-0.30**	-0.32**	-0.20*	-0.37**	-0.35**	-0.16	0.29**	0.33**	-0.34**	-0.17
R_{CT}	0.25*	-0.27**	-0.43**	-0.18	0.00	0.53**	-0.06	-0.14	-0.27**	-0.15
R_{ALP}	0.25*	-0.42**	-0.43**	-0.10	-0.06	0.04	-0.02	-0.04	-0.56**	-0.34**

Note: "*", "**", and "***" means $p < 0.05$, $p < 0.01$, and $p < 0.001$, respectively.
